# Bi-Functional Chicken Immunoglobulin-Like Receptors With a Single Extracellular Domain (ChIR-AB1): Potential Framework Genes Among a Relatively Stable Number of Genes Per Haplotype

**DOI:** 10.3389/fimmu.2019.02222

**Published:** 2019-09-18

**Authors:** El Kahina Meziane, Nicola D. Potts, Birgit C. Viertlboeck, Hanne Løvlie, Andrew P. Krupa, Terry A. Burke, Stewart Brown, Kellie A. Watson, David S. Richardson, Tommaso Pizzari, Thomas W. Göbel, Jim Kaufman

**Affiliations:** ^1^Department of Pathology, University of Cambridge, Cambridge, United Kingdom; ^2^Department of Veterinary Sciences, Institute for Animal Physiology, Ludwig Maximillian University, Munich, Germany; ^3^Department of Physics, Chemistry and Biology, IFM Biology, Linköping University, Linköping, Sweden; ^4^Department of Animal and Plant Sciences, Western Bank, University of Sheffield, Sheffield, United Kingdom; ^5^Aviagen Ltd, Midlothian, United Kingdom; ^6^The Roslin Institute and R(D)SVS, University of Edinburgh, Midlothian, United Kingdom; ^7^Centre for Ecology, Evolution and Conservation, School of Biological Sciences, University of East Anglia, University of East Anglia, Norwich, United Kingdom; ^8^Department of Zoology, Edward Grey Institute, University of Oxford, Oxford, United Kingdom; ^9^Department of Veterinary Medicine, University of Cambridge, Cambridge, United Kingdom

**Keywords:** leukocyte receptor complex, LRC, Fc receptor, FcR, KIR, LILR, avian, reference strand-mediated conformational analysis

## Abstract

The leukocyte receptor complex (LRC) in humans encodes many receptors with immunoglobulin-like (Ig-like) extracellular domains, including the killer Ig-like receptors (KIRs) expressed on natural killer (NK) cells among others, the leukocyte Ig-like receptors (LILRs) expressed on myeloid and B cells, and an Fc receptor (FcR), all of which have important roles in the immune response. These highly-related genes encode activating receptors with positively-charged residues in the transmembrane region, inhibitory receptors with immuno-tyrosine based motifs (ITIMs) in the cytoplasmic tail, and bi-functional receptors with both. The related chicken Ig-like receptors (ChIRs) are almost all found together on a microchromosome, with over 100 activating (A), inhibitory (B), and bi-functional (AB) genes, bearing either one or two extracellular Ig-like domains, interspersed over 500–1,000 kB in the genome of an individual chicken. Sequencing studies have suggested rapid divergence and little overlap between ChIR haplotypes, so we wished to begin to understand their genetics. We chose to use a hybridization technique, reference strand-mediated conformational analysis (RSCA), to examine the ChIR-AB1 family, with a moderate number of genes dispersed across the microchromosome. Using fluorescently-labeled references (FLR), we found that RSCA and sequencing of ChIR-AB1 extracellular exon gave two groups of peaks with mobility correlated with sequence relationship to the FLR. We used this system to examine widely-used and well-characterized experimental chicken lines, finding only one or a few simple ChIR haplotypes for each line, with similar numbers of peaks overall. We found much more complicated patterns from a broiler line from a commercial breeder and a flock of red junglefowl, but trios of parents and offspring from another commercial chicken line show that the complicated patterns are due to heterozygosity, indicating a relatively stable number of peaks within haplotypes of these birds. Some ChIR-AB1 peaks were found in all individuals from the commercial lines, and some of these were shared with red junglefowl and the experimental lines derived originally from egg-laying chickens. Overall, this analysis suggests that there are some simple features underlying the apparent complexity of the ChIR locus.

## Introduction

Many genes that encode type 1 transmembrane (TM) proteins with one to three extracellular C2-type immunoglobulin-like (Ig-like) domains are located together on human chromosome 19, in a region that has come to be known as the leukocyte receptor complex (LRC). These genes in humans include the killer cell Ig-like receptors (KIRs), the leukocyte Ig-like receptors (LILRs), and a receptor that binds to the C-terminal region of certain antibodies (FcR), among others. In general, these receptors either confer inhibition by having a long cytoplasmic tail with an immuno-tyrosine inhibitory motif (ITIM), or confer activation by having a positively-charged amino acid in the TM region that allows interaction with other TM proteins with cytoplasmic tails bearing immuno-tyrosine activating motifs (ITAMs). Cells that bear these receptors integrate the positive and negative signals from these and other receptors to decide on developmental and effector functions [reviewed in (1)].

KIR molecules are expressed on natural killer (NK) cells as well as invariant NK T (iNKT) cells, with the inhibitory KIRs binding classical class I molecules of the major histocompatibility complex (MHC) as ligands, while the activating KIRs bind a variety of ligands typically related to class I molecules, encoded by either host or pathogens. The KIR genes are highly polymorphic, and there are variable numbers of KIR genes in different haplotypes (that is, copy number variation, or CNV), which overall leads to great complexity (although there are a few KIR genes that are found in most or all haplotypes, which have been called “framework” genes). The MHC class I genes that are recognized by KIRs are also highly polymorphic, and the level of interaction between these receptors and their ligands (at the genetic level called epistasis) has enormous effects on the immune response to infectious pathogens and cancer, on autoimmunity and on reproduction [reviewed in (2, 3)].

There are many LILR genes, but only a single FcR gene in the human LRC. LILR molecules (previously known as CD85, LIR, and ILT, and including LAIR1) are expressed on cells of the myeloid lineage, including macrophages and dendritic cells (DCs), as well as some lymphocytes. Different LILRs bind a range of host molecules related to class I molecules, both classical and non-classical, both with and without the small subunit β_2_-microglobulin, and even a pathogen molecule. LILRs are also polymorphic with CNV, and the various interactions with ligands have been reported to strongly affect various immune functions. The Fcα/μR molecule (also known as FCAR and CD89) is expressed in humans on a wide (but not universal) set of myeloid cells, including neutrophils, eosinophils, monocytes, and activated macrophages, some kinds of B cells, follicular dendritic cells (FDCs) and Paneth cells of the intestine, binding IgM and, at lower affinity, IgA antibodies [reviewed in (4–7)].

The equivalent of the LRC in chickens contains over 100 chicken Ig-like receptors (ChIRs) in any particular chicken, which are interspersed over 500–1,000 kbp covering much of microchromosome 37 [reviewed in (8, 9)]. These genes include activating (A), inhibitory (B), and bi-functional (AB) genes, bearing either one or two extracellular Ig-like domains (10–14). Comparison of ChIR sequences, identified from cDNA or genomic DNA (gDNA), has shown almost no overlap between different lines of chickens, suggesting rapid evolution and divergence (15). Certain ChIR-A and ChIR-B molecules have been examined in some functional detail (16, 17). However, many of the ChIR genes encode molecules with both a positively-charged amino acid in the TM region and a long cytoplasmic tail bearing an ITIM, and many of these encode FcRs that bind the chicken antibody isotype IgY (15, 18, 19). An X-ray crystal structure for one of these FcRs has been reported (20).

Given the enormous importance of the LRC genes in human immunity and reproduction, as well as the fascinating epistasis between the LRC and the MHC, we wished to begin a genetic analysis of this region in chickens, which as birds diverged from mammals over 300 million years ago. Moreover, it was reported that aspects of reproduction in a population of red junglefowl were correlated with the MHC class I locus BF1, which was suspected and has since been confirmed to be a ligand for NK cells (21–23). At the time this project was initiated, we were using a hybridization technique to analyse MHC gene polymorphism (24, 25), so we extended that approach to a subset of the ChIR genes, the ChIR-AB1 genes.

## Materials and Methods

### Experimental gDNA Samples

gDNA samples were previously collected (26) from the experimental chicken lines 0, 6_1_, 7_2_, C-B4, C, WL, 15I, P2a, and N that were bred and maintained at the Institute for Animal Health at Compton, UK, and are currently available from the National Avian Resource Facility, Roslin Institute, University of Edinburgh, UK. gDNA samples were also previously collected (26) from lines H.B15, H.B19, H.B21, and CC that were bred and maintained for the Basel Institute for Immunology, Basel, Switzerland. The first three lines were MHC homozygous birds originally from University of Copenhagen, Denmark and are probably not in existence anymore, while the CC line is a highly inbred line still available from the Institute for Molecular Genetics of the ASCR, Prague, Chechia. gDNA was extracted from red blood cells using a modified NaCl protocol (26). gDNA was also provided from individuals of a commercial broiler line and families (mostly trios) of a different broiler line from a major breeding company, as well as from red junglefowl used for previous experiments (21), which at that time were kept at Skara as part of the Swedish University of Agricultural Sciences, and are now maintained at Linköping University in Sweden. All gDNA samples were diluted in Tris-EDTA (TE) buffer (27), and concentrations determined using NanoDrop™ ND-1000 spectrometer.

### PCR

For amplification of exon 2 ChIR-AB1 genes, 25 ng of gDNA was added to a total of 25 μl containing a final concentration of 1 unit VELOCITY DNA polymerase (Bioline), 4 mM deoxyribonucleotide triphosphates (dNTPs, Invitrogen), and 0.4 μM of each primer, UC396 (CCAGCCAGGGGGTGTCSMTG) and UC397 (GTCASCAMCAGCTCCACRGG, where S is G and C, M is A and C, and R is G and A), and PCR carried out on a DNA Tetrad 2 Thermal Cycler (Biorad) with denaturing at 96°C for 1 min, 30 cycles of denaturation at 96°C for 1 min, annealing at 63°C for 30 s, and extension at 72°C for 1 min, and a final extension at 72°C for 10 min. For production of fluorescently-labeled reference strands (FLRs), the same general conditions were used, except the template was 25 ng of a ChIR-AB1 cDNA in a pcDNA3 plasmid, with the 5′-FAM (carboxy-fluorescein) labeled reverse primer UC485 (GTCAGCACCAGCTCCACGGG, MWG Biotech AG, Germany) at a final concentration of 4 μM but the unlabeled forward primer UC483 (CCAGCCAGGGGGTGTCCCTG) at a final concentration of 0.4 μM, to generate an excess of fluorescent reverse strand FLR. The amplified FLR preparations were purified using QIAGEN PCR purification kit (following the manufacturer's protocol), examined by electrophoresis at 140 V for 30 min by a 0.8% agarose gel in TAE (27) containing 5 μl/ml GelRed (Biotium) to check purity, and stored in TE at 4°C in the dark.

### Cloning, Sequencing, and Analysis of Amplicons

gDNA from a line 6_1_ chicken was subjected to PCR using the degenerate primers as described above, the amplicons were purified with the QIAGEN PCR purification kit, concentrated by ethanol precipitation (27), and ligated into a pJET vector using the blunt-end protocol described in the CloneJET™ PCR Cloning Kit (Fermentas). By standard procedures (27), chemically-competent DH5α E. coli bacteria were transformed, plated on LB agar plates containing 100 μg/ml ampicillin and incubated at 37°C overnight; multiple colonies were picked the next day and overnight cultures grown for miniprep DNA (QIAGEN). Dideoxy-chain termination (Sanger) sequencing on one strand (from T7 primer) was performed (DNA sequencing facility, Department of Biochemistry, University of Cambridge), and sequences were curated using Bioedit [http://www.mbio.ncsu.edu/BioEdit/bioedit.html, (28)]. Sequence alignments [including ChIR-AB1 sequences from chicken lines M11 and R11 (15)] were made using Clustal (www.ebi.ac.uk/Tools/msa/clustalo/) with phylogenetic trees visualized using dendroscope (http://dendroscope.org/), or by MAFFT and trees displayed using Archaeopteryx.js (https://mafft.cbrc.jp/alignment/server/). The 39 sequences were deposited in GenBank with accession numbers MK605290 to MK605326 (for EM1T7-1 to EM21T7-2).

### RSCA

Analysis based on the original method (29) was extensively optimized (24, 25). In brief, amplification of FLRs and of experimental samples was carried out as described above, and then the amplicons of experimental samples were hybridized with FLR, chromatographed, and analyzed. Prior to hybridization, amplified FLRs were diluted in 1:40 with PCR grade water. In a 96-well plate (Star Labs), 3 μl of amplified FLR diluted 1:40 with PCR grade water was mixed with 4 μl of each genomic PCR product, and the samples hybridized in a DNA Tetrad 2 Thermal Cycler (Biorad) by denaturation at 95°C for 10 min, ramping down to 55°C at 1°C/sec, re-annealing at 55°C for 15 min, and cooling to 4°C for 14 min. Hybridized samples were diluted 1:2 with PCR grade water, the carboxy-X-rhodamine (ROX) fluorescently-labeled size standard GeneScan ROX 2500 (Applied Biosystems) was diluted 1:50 with PCR grade water, and 2 μl of each were added to each well of a 96-well semi-skirted PCR plate (Star Labs). Each plate included wells of size standard alone and FLR hybridized with water, to control for FLR purity and size standard quality. The plates were run on an automated capillary sequencer (ABI 3100 Prism Genetic Analyzer) under non-denaturing conditions at the NERC Biomolecular Analysis Facility (NBAF) at the University of Sheffield, using an injection voltage of 8 kV/30 s. The output was analyzed by Genemapper software (Applied Biosystems), with each fluorescent peak distinguished by a time value compared to the size standard peaks.

## Results

### RSCA and Sequencing of ChIR-AB1 Extracellular Exon Give Two Groups of Peaks With Mobility Correlated With Sequence Relationship to the FLR

The literature showed the bi-functional ChIR genes that encode a single extracellular Ig-like domain, the ChIR-AB1 genes, had three desirable characteristics for RSCA (13–15). First, there is a moderate number of genes reported, ranging from 15 to 23 in individual chickens based on cDNA and genomic sequences. Second, the ChIR-AB1 genes are interspersed throughout the available genomic sequences of the chicken LRC, so that changes anywhere in the LRC should impact the RSCA patterns of the ChIR-AB1 genes. Third, exon 2, encoding the single extracellular Ig-like domain, is a suitable length for amplification by PCR so that intron lengths would not be an issue. A large number of cDNA and gDNA sequences from the chicken lines M11 and R11 (15) were compared to find suitably conserved short sequences from which to design oligonucleotide primers that would bind to every ChIR-AB1 gene, with the ones chosen amplifying a fragment of 230 nucleotides, with the central 190 nucleotides corresponding to the gene sequence between the primers.

gDNA was isolated from erythrocytes of line 6_1_ chickens, a well-characterized experimental line with the MHC haplotype B2. After amplification, cloning and sequencing, 39 line 6_1_ sequences (Figure S1) were compared with 36 previously published sequences from lines M11 and R11 (MHC haplotypes B2 and B15) by a neighbor-joining (NJ) phylogenetic tree, showing that the new clones are diverse and, as expected from previous reports (15), are not strongly overlapping with previously described ChIR-AB1 sequences. Indeed, many of the sequences from line 6_1_ chickens are on distinct clades distant from the M11 and R11 sequences (Figure S2).

Using a cDNA clone derived from an M11 chicken (15), a fluorescently-labeled reference strand (FLR) in the negative sense (called FLR29) was produced by amplification in PCR for which the reverse primer was coupled to carboxy-fluoresceine (FAM) and was present at 10 times the concentration of the untagged forward primer. The gDNA samples were amplified with the same primers without tags and at the same concentration, and the FLR and experimental amplicons mixed, boiled, and cooled to allow hybridization. The hybridization mix was added to fluorescently-labeled standards and analyzed by non-denaturing capillary electrophoresis, producing outputs with peaks detected by fluorescence, the first of which being a homoduplex of the FLR with the complementary strand, and the rest being heteroduplexes of the FLR with complementary strands amplified from the gDNA sample.

**Figure 1:**
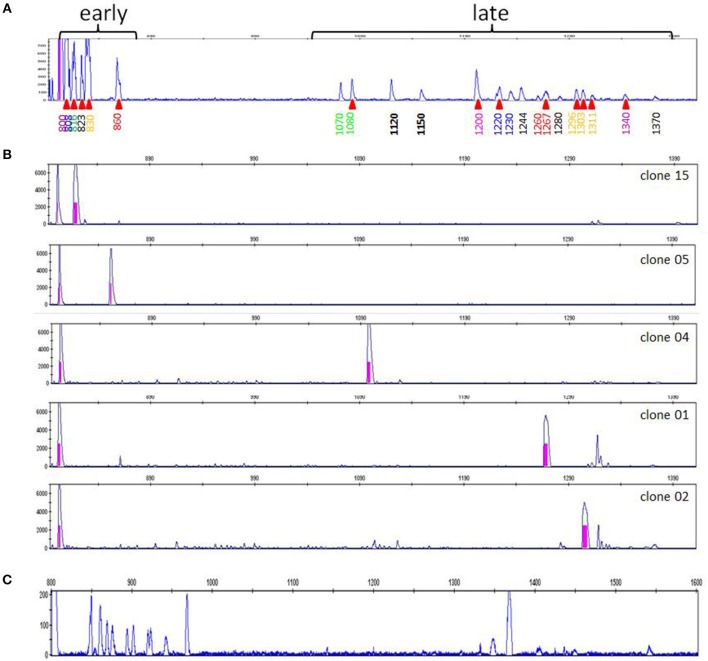
RSCA patterns of ChIR-AB1 amplicons using two FLRs reveal two groups of sequences, with the patterns arising from combination of single peaks from individual sequences. **(A)** RSCA pattern of line 6_1_ gDNA using FLR29, with elution time indicated for each peak along the x-axis and amplitude on the y-axis. Red triangles under the elution times indicate those peaks for which individual DNA clones were isolated and tested by RSCA. **(B)** RSCA patterns of four cloned amplicons (others not shown) from line 6_1_ gDNA using FLR29 show that individual clones give single peaks that have similar elution times to peaks in the original pattern, with mixing experiments used to show identity (data not shown). The fact that repeated RSCA analysis of single cloned sequences consistently showed the same single peaks demonstrates that, under the PCR conditions used, only one sequence was amplified despite the use of degenerate primers for amplification. **(C)** RSCA pattern of line 6_1_ gDNA using FLR22, which spreads out the peaks but still shows two groups of sequences (although perhaps not the same groups).

**Figure 2:**
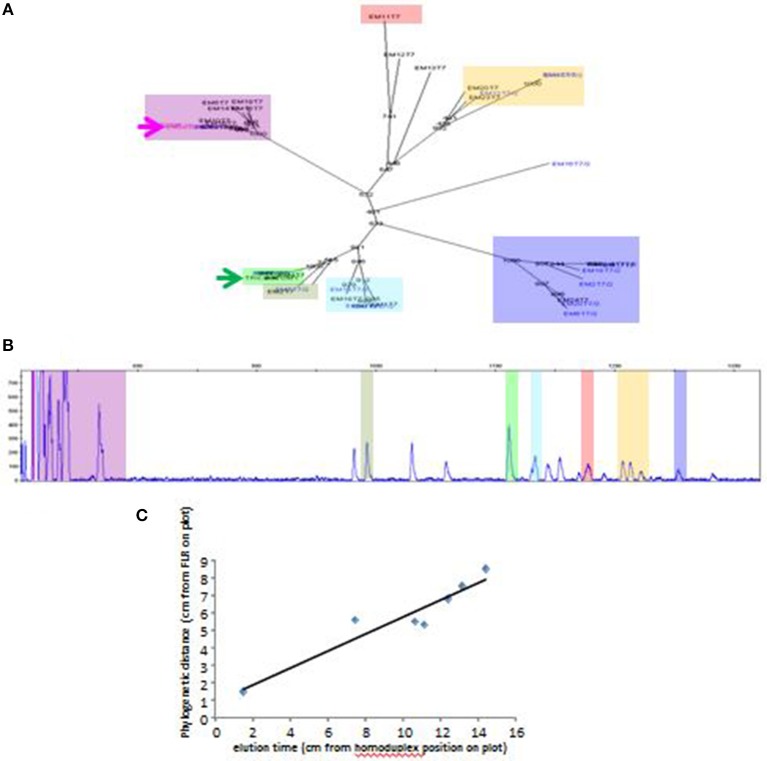
Elution time of peaks from RSCA pattern of ChIR-AB1 amplicons from line 6_1_ gDNA using FLR29 correlates roughly with phylogenetic distance between cloned sequences. **(A)** Phylogenetic tree of sequences, with FLR29 indicated with a pink arrow and a FLR22 indicated with a green arrow, and with different clades highlighted with different colors. **(B)** RSCA pattern similar to the one in Figure 1A but with location of cloned sequences highlighted using the same color scheme as in the phylogenetic tree. **(C)** Plot of phylogenetic distance (as measured by distance along the branches of the tree from FLR29) and elution time (as measured by distance on the chromatographic trace from the position of the homoduplex).

A typical RSCA output from a line 6_1_ chicken yielded one group of heteroduplex peaks close to the homoduplex peak, and another group eluting much later (Figure 1A). RSCA using the cloned line 6_1_ amplicons gave single heteroduplex peaks, each of which was at the position of one of the peaks from the whole line 6_1_ pattern (some examples shown in Figure 1B). Comparing the sequences with the RSCA showed that early heteroduplex peaks were in the same clade as the FLR sequence in the phylogenetic tree, while later peaks were found on branches further away (Figure 2). Later in the project, a second FLR (FLR22) from a clade different from the first FLR was produced, which gave two groups of peaks, but a different number and pattern from FLR29 (Figure 1C), highlighting the need to use multiple FLRs to ensure the most complete results. Overall, these investigations showed that RSCA gives a pattern that faithfully reflects the diverse sequences and therefore can be used for genetic analysis.

### RSCA of ChIR-AB1 Extracellular Exon Shows Simple Peak Patterns for Experimental Chicken Lines but Much More Complex Patterns for a Commercial Broiler Line and a Red Junglefowl Flock

**Figure 3:**
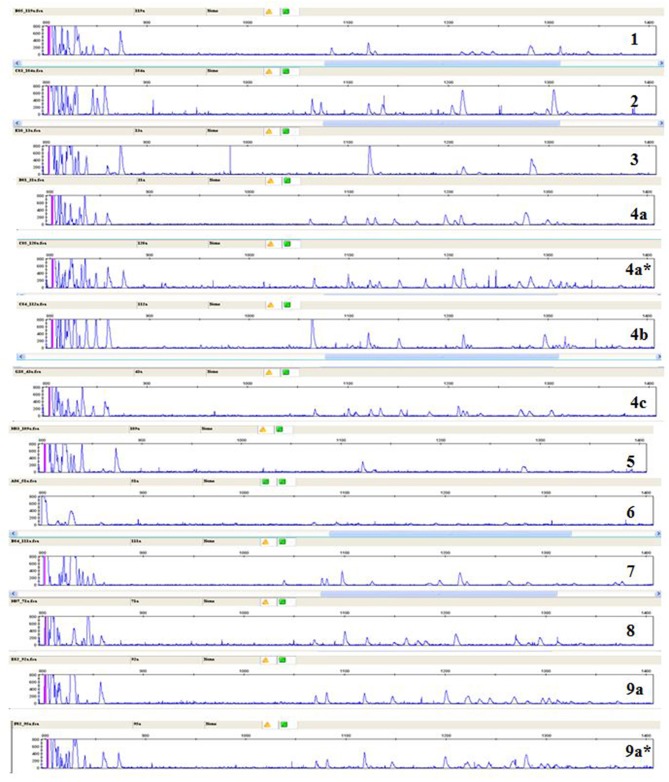
RSCA of ChIR-AB1 amplicons from experimental chicken lines using FLR29 shows nine major patterns (indicated by numbers) and a few frequent variants of those patterns (indicated by letters and stars), as determined by visual inspection.

**Figure 4:**
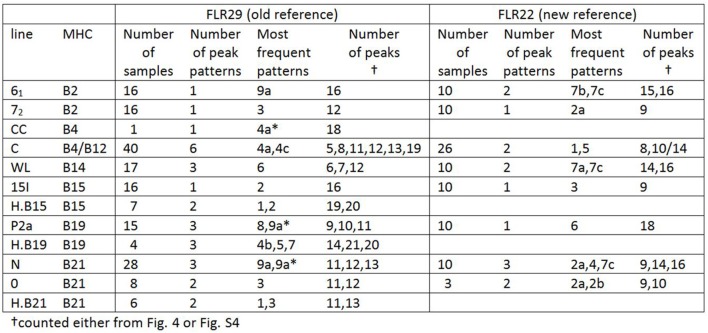
Tabulation of results from RSCA analysis of experimental chicken lines, showing that most lines have one or a few patterns, with comparable but slightly different results for the two FLRs (based on Figures 3, 5 and Figure S4).

**Figure 5:**
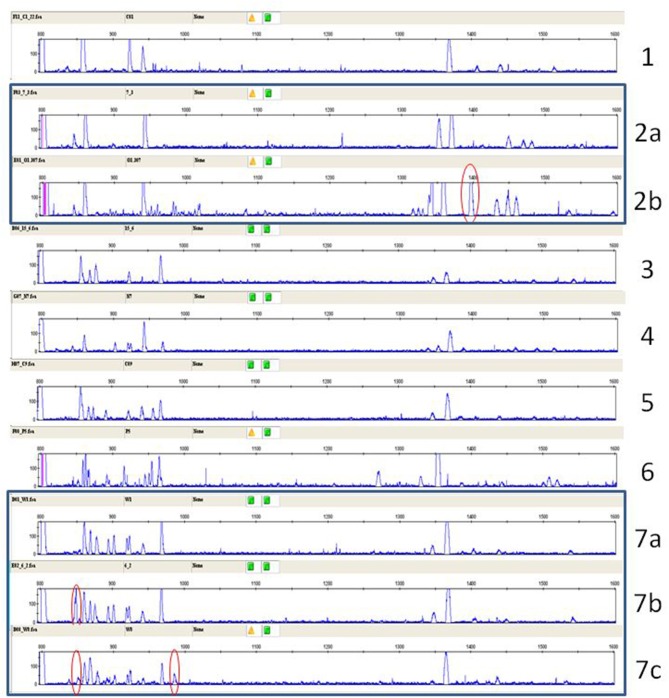
RSCA of ChIR-AB1 amplicons from experimental chicken lines using FLR22 shows seven major patterns (indicated by numbers) and a few frequent variants (indicated by letters in the identifier, and boxed together), as determined by visual inspection. Peaks that differentiate the variants are circled in red.

A few relatively inbred experimental chicken lines derived from commercial egg-type (layer) chickens, typically with known MHC haplotypes, have been the mainstay of avian immunology and disease research for many decades (30), so a collection of gDNA from such experimental lines maintained in the UK and previously available in Switzerland (26) was examined. RSCA with FLR29 found nine major ChIR-AB1 patterns of peaks (and some variants) at high frequency found in 12 such lines (Figure 3), with the patterns highly reproducible between individuals (Figure S3). The numbers of peaks range from roughly 1 to 13 in the group far from the homoduplex (and depending on what amplitude of a peak is chosen as a cutoff), while the numbers near the homoduplex are relatively difficult to discern, but ranging perhaps from 3 to 9 (Figure 3; Table S1). Using FLR29, four of the 12 lines have only a single pattern in all individuals, three lines have two patterns, four lines have three patterns and one line has six patterns (Figure 4). The four highly inbred lines (CC, 6_1_, 7_2_, 15I) have the fewest patterns, but it seems likely that the number of patterns is only accurate for lines for which many individuals were examined. Somewhat different patterns were found by RSCA with FLR22 (Figure 5), with one line giving a single FLR29 pattern now showing two related FLR22 patterns, and other lines with more than one FLR29 pattern having fewer FLR22 patterns (Figure 4). There appears to be no relationship with the MHC haplotype and the ChIR-AB1 haplotype (Figure 4), as expected since these genomic regions lie on different microchromosomes (12, 31).

**Figure 6:**
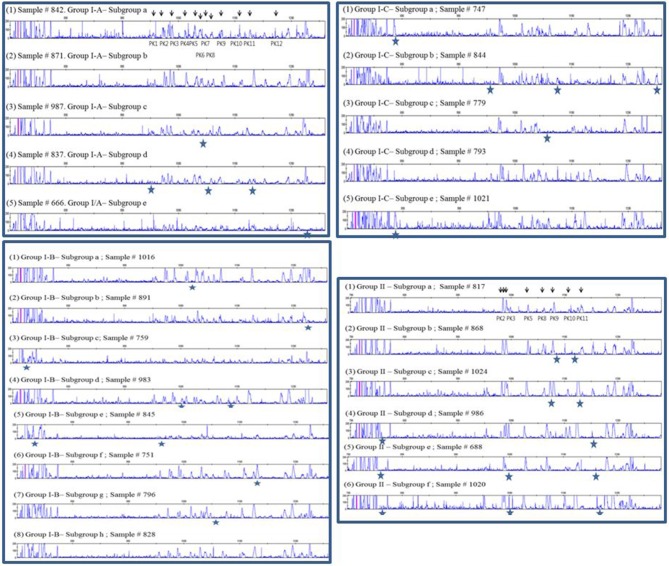
RSCA of ChIR-AB1 amplicons from a commercial broiler line using FLR29 shows 24 patterns by visual inspection that can be subdivided into four groups, three of which are more closely related. Arrows indicate the peaks that were used to determine the groups; stars indicate peaks that might; or might not be present in that pattern.

More relevant to the poultry industry are the huge numbers of commercial meat-type (broiler) chickens, so a single line from a commercial breeding company was examined. Many more peak patterns were found, and all of them had many more peaks than the inbred experimental chickens (Figure 6). Four major groups of patterns were found, three of which were more closely related than the fourth, and many variants were found within each major pattern, leading to 24 patterns discerned by eye (Figure 6; Figure S4). In comparison, this line is known to have four MHC haplotypes, two of which comprise over 90% of genotypes [(25), N. Potts and J. Kaufman, unpublished].

**Figure 7:**
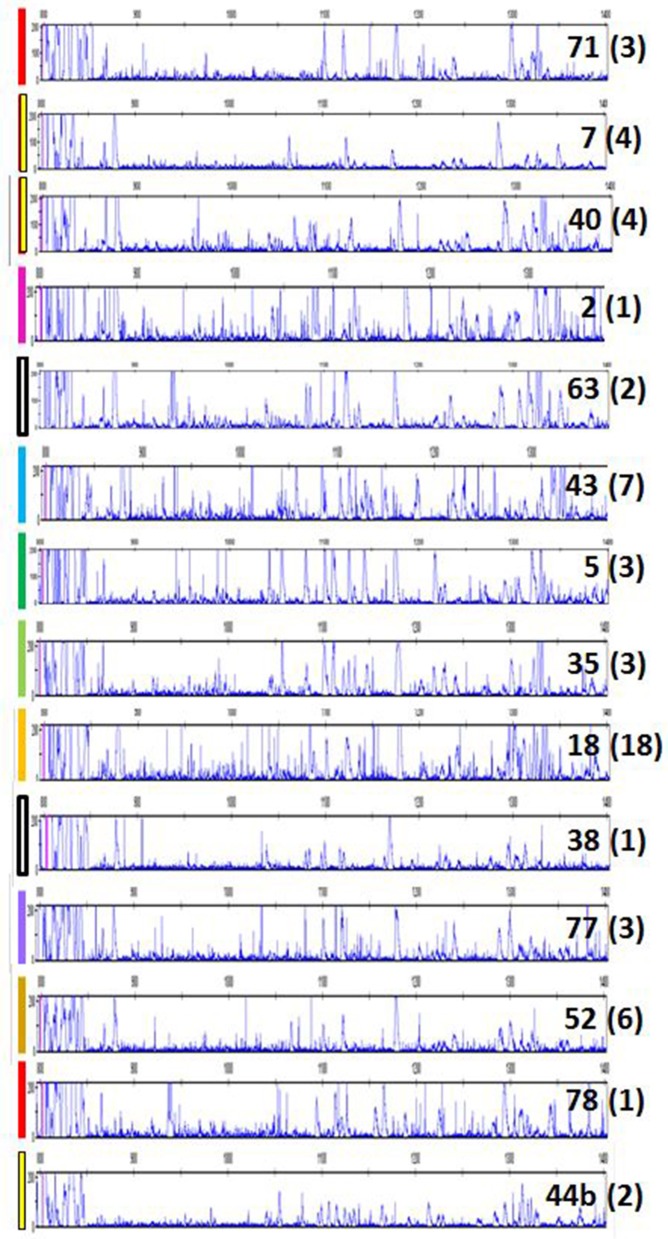
RSCA of ChIR-AB1 amplicons from a red junglefowl flock using FLR29 shows 14 patterns by visual inspection, validated by careful analysis of peaks in Table S2. Colored bars on left indicate the groups indicated on the left of Table S2; each number on the right is the sample number for an individual representative of a group, and in parentheses the number of individuals in that group.

Finally, it was of interest to investigate chickens more closely-related to the presumed ancestors of chickens domesticated between 4,000 and 8,000 years ago, so a flock of red junglefowl originating as wild birds in Thailand was examined. Again, many more peak patterns were found, and all of them had many more peaks than the inbred experimental chickens (Figure 7). A careful curation and comparison of the many patterns from among the 63 individual chickens examined showed that some patterns were very frequent, while others were rare, with some 14 in total (Figure 7; Table S2). Moreover, it appeared that some peaks are shared between many individuals, an observation considered more closely in the next section.

### Complex Peak Patterns in the Commercial Broiler and Red Junglefowl Chickens Are due to Heterozygosity, With Some Peaks Shared Among Many Chickens Examined

**Figure 8:**
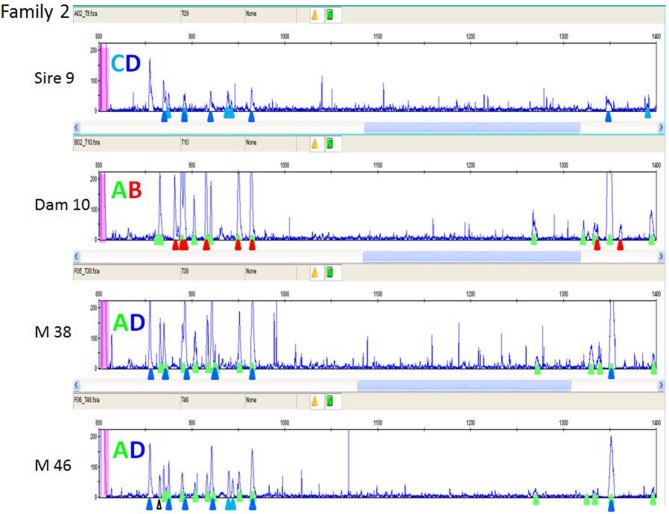
RSCA of ChIR-AB1 amplicons from family 2 of a second commercial broiler line using FLR22, showing that ChIR-AB1 haplotypes can be identified in parents and offspring, along with evidence of recombination. The peaks corresponding to haplotypes C (light blue triangles) and D (dark blue triangles) in sire 9 and the haplotypes A (green triangles) and B (red triangles) from dam 10 are found in the male offspring M38 as clear A and D haplotypes. However, the other male offspring M46 has all the peaks from the maternal A haplotype, and all but one peak from the paternal D haplotype along with two peaks from the paternal C haplotype and one unexpected peak (white triangle), suggesting recombination between paternal haplotypes with creation of a novel sequence.

In order to understand the more complex pattern of peaks found outside of the inbred lines, families from a particular line of commercial broiler birds (dam, sire, one or more offspring) were examined. As mentioned previously, a second reference (FLR22) was developed, the sequence of which is more closely-related to the peaks appearing later in capillary electrophoresis with the original FLR29. RSCA with this new FLR22 also gave two groups of peaks, but with the sequences closer to the new FLR appearing earlier in the run. Moreover, the peaks were overall more evenly spread out, so that identifying the peaks was much easier. One example illustrates these points (Figure 8), and shows that unambiguous haplotypes can be determined from the results of one offspring. However, the peak pattern in the other offspring shows evidence of recombination among the paternal haplotypes by appearance and disappearance of peaks, as well as the appearance of an unexpected peak that might reflect the creation of a new hybrid gene. Examining 15 such families by RSCA allowed unambiguous identification of ChIR-AB1 haplotypes along with loss or gain of individual peaks (further examples in Figure S5), and for all of these haplotypes, the peak pattern was similar in complexity to those in inbred lines.

In light of the results with the commercial families, it became apparent that a few of the peak patterns for red junglefowl were simpler than the others and might represent homozygotes: the yellow group including sample 71, the red group including sample 7 and a potential recombinant between the two which is sample 2 (Figure 7; Table S2). All the other patterns might be explained as heterozygotes between these simpler patterns, along with peaks that appear or disappear presumably due to recombination. Therefore, the complex patterns found in the commercial chickens and the red junglefowl are likely to be due mostly to heterozygosity (with occasional recombination and other processes), suggesting that the number of ChIR-AB1 genes in different haplotypes is relatively stable.

**Figure 9:**
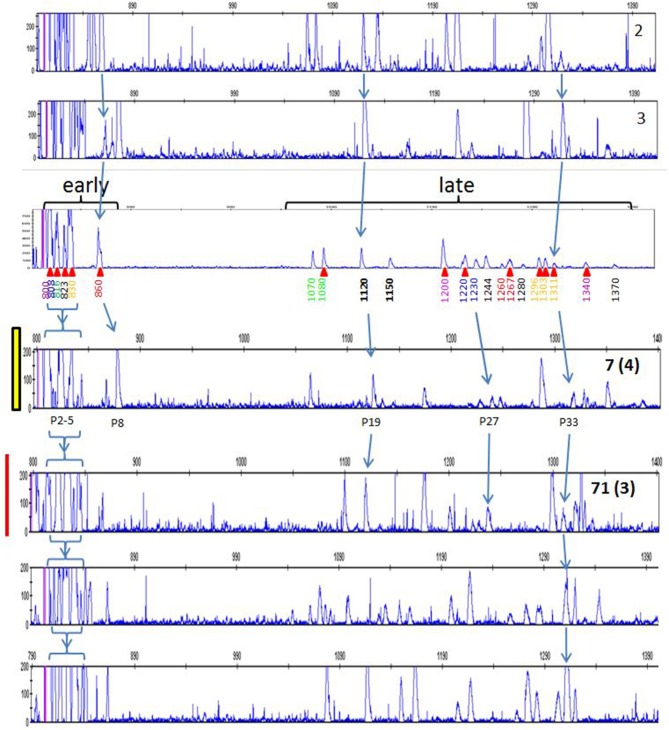
RSCA of ChIR-AB1 amplicons from various chickens using FLR29, top to bottom: two samples from experimental chicken haplotypes 2 and 3 (as defined in Figure 5), the same panel a from Figure 1 depicting haplotype 9a from experimental line 6_1_ including elution times and red arrows indicating cloned sequences, the same two panels for individuals 7 and 71 from Figure 7 representing to two putative homozygote haplotypes, and two randomly chosen samples from the first commercial broilers line. Arrows indicate peaks thought to be the same between panels, with the peak numbers from Table S2 depicted below the panel for red junglefowl 7.

**Figure 10:**
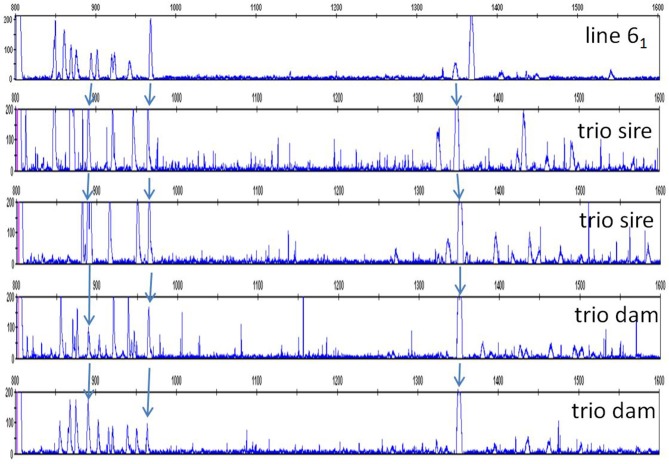
RSCA of ChIR-AB1 amplicons from various chickens using FLR22, top to bottom: the same panel C from Figure 1 depicting haplotype 9a from experimental line 6_1_, two randomly chosen sires and two randomly chosen dams of families from the second commercial broiler line. Arrows indicate peaks thought to be the same between panels.

Careful consideration of the peaks in the red junglefowl showed that some peaks were found in many if not all of the individuals (Table S2), and comparison of the patterns with those of the inbred lines and the first commercial line (using FLR29) identified a number of peaks that were widely shared (Figure 9). Using FLR22, which spreads out the peak patterns for easier identification, a few peaks were shared among the families of the second commercial line, as well as with some of the inbred lines (Figure 10). A few mixing experiments (as in Figure S6) confirmed the co-location of peaks in the samples examined, but much more work is required to confirm these tentative identifications. However, the existence of these shared peaks suggests that the cloning experiments may have underestimated the number of shared genes between ChIR haplotypes, pointing to the possibility of framework ChIR genes that are widely conserved along with other ChIR genes that are found more rarely.

## Discussion

The genes of the LRC in humans play crucial roles in biology, discovered chiefly by genetics. In particular, interactions between the highly polymorphic MHC class I molecules and the polymorphic polygenic KIRs and LILRs lead to striking epistasis, with effects on two of the strongest and most rapid selective forces in evolution: immunity and reproduction (2). In order to begin to unravel the potential interactions of the related genes in chickens, a relatively quick and high through-put analysis system was developed for classical MHC genes (24, 25) and for ChIRs. Of the various kinds of ChIR genes, the ChIR-AB1 genes were chosen because of their simplicity, limited numbers, and spread throughout the LRC.

This work shows that RSCA is a suitable method for analyzing ChIR-AB1 genes, giving clear patterns that are highly reproducible between individuals within a single analysis run, even with samples that have many peaks. However, there are several difficulties with this approach (25). First, it is somewhat cumbersome and has required significant optimization of many of the steps. Second, reproducibility between runs is reasonable but not perfect; the positions of peaks relative to the fluorescent standards varied slightly between runs. Third, the amplitudes of peaks with the same apparent position vary between samples, so it is not clear whether there are experimental differences in the amplification or hybridization, differences in the copy number of the same gene, or location of different sequences at the same position. This became a serious problem with the highly complex samples, with the number of peaks identified depending on the overall amplitude found within the particular run. Fourth, in order to understand the molecular basis of the peaks, additional cloning and sequencing would be necessary. However, a significant advantage of such a hybridization technique compared to sequencing is a relative insensitivity to low level contamination (25).

**Figure 11:**
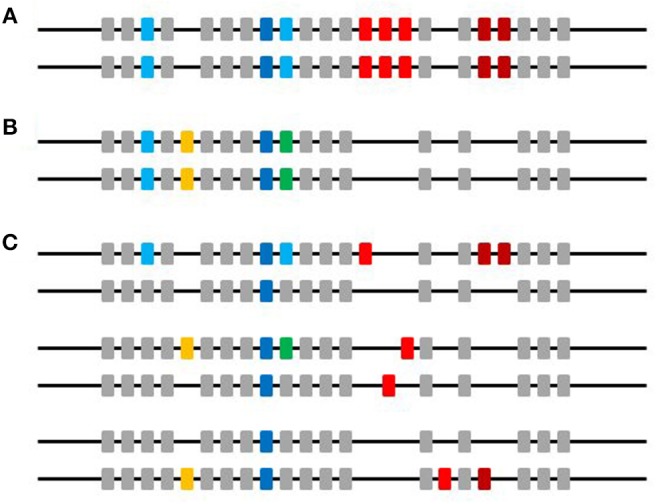
A picture to summarize the data in this paper. Horizontal line indicates microchromosome 37 and boxes indicate genes, with colors for different ChIR-AB1 alleles and loci, and gray for other ChIR families. **(A)** The two chromosomes present in an individual of an inbred strain. **(B)** The two chromosomes present in an individual of another inbred strain. **(C)** The two chromosomes present in three individuals of an outbred population. The individuals within an inbred strain are mostly homozygotes for the ChIR-AB1 loci, while the individuals in the populations of commercial birds and red junglefowl are mostly heterozygotes. ChIR-AB1 loci are scattered throughout the chromosome, with non-homologous recombination and deletion giving rise to haplotypes with significant CNV. At least one locus is found in every bird examined (dark blue boxes), and other loci are found in many lines and individuals from outbred populations (light blue and yellow boxes). Other loci are found less frequently (green and dark red boxes). There is in fact no evidence from the work in this paper that the genes giving rise to peaks representing a unique sequence are actually located at the same genomic location in different chromosomes (red boxes).

The results in this report are just the very first steps of analyzing the complex ChIR locus at a genetic level, but they point to both underlying simplicity and further complication that need to be further examined (Figure 11). First, there appear to be (at least) two kinds of ChIR-AB1 sequences amplified, those with close sequence identity to the original FLR29 and those that are not so close. Results from the use of the second FLR22 showed that this grouping into two kinds of sequences is robust. Since phylogenetic trees of amplified sequences from line 6_1_ suggest more than two clades, this result is most easily explained by significant sequence differences between clades, so that the RSCA separates peaks into those sequences relatively close to the FLR vs. those that are farther away.

Second, the simplicity and low numbers of peak patterns found in the experimental lines suggests that each of these lines has only one or a few ChIR-AB1 haplotypes, presumably due to the inbreeding process. However, one might have expected recombination to scramble the peak patterns in those lines with multiple haplotypes, but the level of different variants was very modest, so perhaps the level of recombination within the LRC covered by the ChIR-AB1 loci is not great. Even for the individuals from lines with a single haplotype, one might have expected copy number variation (CNV) from unequal crossing-over or deletion between similar genes within a large multigene family, and this also was not frequent in the inbred lines. The numbers of peaks within a haplotype ranged up to 20, which is close to what one might expect from previous studies (15), if the birds examined in those studies were in fact heterozygotes.

Third, the very complex patterns of peaks found for commercial broiler chickens and red junglefowl resolve into independently segregating haplotypes, by analysis of families for the commercial broiler chickens and by inspection of peak patterns for red junglefowl, with a similar number of peaks found as in the haplotypes from inbred chickens. This realization reduces both the number of potential genes within an individual (since the peaks come from two different haplotypes) and the number of haplotypes found in a population (since many of the peak patterns are the result of the same haplotype found as a heterozygote with several other haplotypes). However, it cannot be ruled out that the PCR primers designed on the basis of sequences from experimental chicken lines might fail to amplify all ChIR-AB1 family members from commercial chicken lines or red junglefowl. Moreover, this simplification is complicated by the presence of single peaks that appear or disappear from otherwise extremely similar peak patterns (both in the lines and in the families), potentially due to unequal crossing over or deletion, as mentioned in the previous paragraph.

Fourth, some peaks in apparently the same position were shared widely among birds from different inbred strains, from commercial birds and even from the red junglefowl. Such a result might not be expected from the cDNA and gDNA sequencing carried out thus far, in which extremely few ChIR sequences were shared between different chicken lines. Without further analysis, it is not clear that these peaks represent the same sequence, but this finding was a surprise and deserves further investigation.

The number and complexity of the peak patterns found in the commercial broiler line and red junglefowl proved to be too much to allow unraveling of potential interaction with other loci such as the MHC (E. K. Meziane and J. Kaufman, unpublished). However, the simplifications suggested from this study might eventually be harnessed to allow such genetic analysis to be performed. The use of a hybridization technique like RSCA has been superseded by next generation sequencing (NGS), which can be faster, higher through-put and more informative. Such an NGS typing system for chicken classical MHC genes has been built on the basis of earlier RSCA studies, and replicates the previous findings while extending the reach of the analysis (C. A. Tregaskes and J. Kaufman, unpublished). Developing such an NGS typing system for ChIR genes seems like a logical next step in attempting to understand this complex system and its interactions with other genetic loci.

## Data Availability

The datasets generated for this study can be found in GenBank, MK605290 to MK605326.

## Resources

The 39 ChIR-AB1 sequences from gDNA of line 6_1_ were deposited in GenBank with accession numbers MK605290 to MK605326 (for EM1T7-1 to EM21T7-2).

## Author Contributions

JK conceived the study and wrote the paper. EM carried out all of the experiments with the aid and advice of NP, AK, and TB. BV and TG provided unique clones and sequences. HL, SB, KW, DR, and TP provided unique samples. JK and EM made the figures. All authors had the opportunity to comment on the draft.

### Conflict of Interest Statement

At the time of this research, KW was employed by company Aviagen Ltd., and SB continues to be employed by Aviagen Ltd. The remaining authors declare that the research was conducted in the absence of any commercial or financial relationships that could be construed as a potential conflict of interest.

## Supplementary Material

Figure S1 Sequences of 39 clones of 190 base pairs from two independent amplifications of exon 2 from a line 6_1_ chicken, plus the two FLRs.

Figure S2 Neighbor joining tree of 36 cloned sequences from Munich lines M11 and R11 (all indicated by names starting with S), and 39 sequences from a line 6_1_ chicken (all indicated by names starting with EM), plus the two FLRs (indicated by arrows, orange for FLR22, and green for FLR29). The tree was constructed using MAFFT (https://mafft.cbrc.jp/alignment/server/) displayed using Archaeopteryx.js available on the same website.

Figure S3 RSCA of ChIR-AB1 amplicons using FLR29 show that patterns can be very reproducible between different individuals within a class of patterns on the same run. **(A)** Three individuals from line 15I (pattern 2), **(B)** four individuals from line C-B4 (pattern 4c), **(C)** three individuals from the first commercial broiler line.

Figure S4 RSCA of ChIR-AB1 amplicons from one group of commercial broiler chickens using FLR29 shows peaks that appear or disappear. Purple highlight indicates peaks common to all patterns shown, yellow highlight and star indicate peaks shared with pattern I/A-1, green highlight and plus sign indicate peaks not shared with pattern I/A-1.

Figure S5 Families (trios) in a line of commercial birds allow haplotypes to be discerned by analysis of RSCA patterns using FLR22.

Figure S6 RSCA with mixed samples identifies peaks with the same mobility, confirming identical peaks. Two samples with different peak patterns (3 and 4c) were analyzed either separately or mixed together (half and half to give the same loading volume). The shaded peaks that appear to have the same mobility when analyzed separately are confirmed to collapse to a single peak, while other peaks remain unchanged.

Table S1 Spreadsheet of peaks from RSCA using FLR29 for the 12 inbred lines.

Table S2 Spreadsheet of peaks from RSCA using FLR29 for a red junglefowl flock, indicating potential homozygotes of possible parental haplotypes, as well as potential framework genes.
