# Supplementary material for: Bi-Functional Chicken Immunoglobulin-Like Receptors With a Single Extracellular Domain (ChIR-AB1): Potential Framework Genes Among a Relatively Stable Number of Genes Per Haplotype
Source: Front Immunol. 2019 Sep 18;10:2222. doi: 10.3389/fimmu.2019.02222 (PMC6760009; doi:10.3389/fimmu.2019.02222)
Supplement: Figure S1 — Sequences of 39 clones of 190 base pairs from two independent amplifications of exon 2 from a line 61 chicken, plus the two FLRs. [file Data_Sheet_1.PDF]

>EM29-396-uc483 (FLR)
TCCCGCATGGCTGCCTGGGTCCAGCTCTGGCTCAATGGAACTCTGAGATTTGACAAGGAAAAAGACAAGGA
GCAGGATGCAGCTGAGTTCTCCTTTGCTGTCACAAACCTGGAGGACGCCGGGACATATCAGTGTCGGTACCA
GGTGTCAGAGCCACTGTGGACATCAAATCAGAGTGACCCCGTGGAG

>EMFLREM22UC483
GCCCCGGCCAGCTACCAGGGTCGAGCTGTATCAGGATGGACAGTGGAGATCCAGAAAGGAAATGGACCAGG
AGCAGGACGTGGCTGAGTTCTCCTTGGCTGGAATAAAGAAGAAAGATTCGGGGACATATCAGTGCCAGTACC
AGGGGTTGGCACCTGCAGGGACATCAGAGAAGAGTGACCCCGTGGAG

>EM1T7-1
GCCCCGGCCGGCTGCCTGGGTTGAGCTGTACTGGGAGGGCAAGTGGAGATTCAGAAAAGAAATGGACCAGG
AGCAGGACGTGGCTGAGTTCTCCTTGACTGGAATTAAGCAGGAAGATGCAGTGAGATATCAGTGCCAGTACC
AGGGGTTGGAGCCTGCAGGGACATCGCAGAAGAGTGACCCCGTGGAG

>EM2T7-1
GCCCCAGCCAGCTGCCTGGGTCGAGCTGTACCAGGATGGACAGTTGAGATCCATAAAGGAAATGGACCGGA
AGCAGGACGTGGCTGAGTTCTCCTTGGCTGGCATAAAGAAGGAAGATTCGGGGACATATCAGTGTCGGTACC
AGGGGTTGGAGCCTGCAGGAACATCACAGAAGAGTGACCCCGTGGAG

>EM3T7-1
GCCCCAGTTGCACTTACAAGTTCAGCTCTGCCAGAACGGACATATGAGATCCAAGAAAGACGCGGAGATGCT
TCAGGACACAGTTGAGTTTTCCTTGGTTAGCGTAGAGAAGGAAGACGCAGAGAAGTATCGGTGCCAGTACCG
GGTTTTGGAGCCACCGGGGACATCAGGGAAGAGTGACCCCGTGGAG

>EM4T7-1
GCCCCAGCCAGCTGCCTGGGTCGAGCTGTACCAGGATGGACAGTTGAGATCCAGCAAGGAAATGGACCAGG
AGCAGGACGTGGCTGAGTTCTCCTTGGCTGGCATAAAGAAGGAAGATTCGGGGACATATCAGTGCCAGTACC
AGGGGTTGGCACCTGCAGGGACATCAGAGAAGAGTGACCCCGTGGAG

>EM5T7-1
GCCCCGCATGGCTGCCTGGGTCCAGCTCTGGCACAATGGAACTCTGAGATTGAAGAAGGAAAAAAACAAGGA
GCAGGATGCAGCTGTGTTCTCCTTTGCTGTCACAAAACTGGAGGACGCCGGGACGTATCAGTGTCGGTACCA
GGTGTCAAAGCCTCTGAGGACATCCAAAAAGAGTGACCCCGTGGAG

>EM7T7-1
GCCCCGGCCGGCTGCCTGGGTTGAGCTGTACAAGGAGGGAAAGTGGAGATCCAGAAAAGAAATGGACCAGG
AGCAGGACGTGGCTGAGTTCTCCTTGACTGGAATTAAGCAGGAAGATGCAGTGAGATATCAGTGCCAGTACG
AGGGGTTGGAGCAACCAGGGACATCGGAGAAGAGTGACCCCGTGGAG

>EM8T7-1
GCCCCGGCCAGCTACCAGGGTCGAGCTGTATCAGGATGGACAGTGGAGATCCAGAAAGGAAATGGACCAGG
AGCAGGACGTGGCTGAGTTCTCCTTGGCTGGAATAAAGAAGAAAGATTCGGGGACATATCAGTGCCAGTACC
AGGGGTTGGCACCTGCAGGGACATCAGAGAAGAGTGACCCCGTGGAG

>EM9T7-1
GCCCCGCATGGCTGCCTGGGTCCAGCTCTGGCACAATGGAACTCTGAGATTTGACAAGGAAAAAGACAAGGA
GCAGGATGCAGCTGAGTTCTCCTTTGCTGTCACAAACCTGGAGGACGCCGGGACATATCAGTGTCGGTACCA
GGTGTCAGAGCCACTGTGGACATCAAATCAGAGTGACCCCGTGGAG

Fig. S1, page 1of 1. Sequences of 39 clones of 190 base pairs from two independent amplifications of exon 2 from a line 6_1 chicken, plus the two FLRs

>EM10T7-1
GCCCCGCATGGCTGCCTGGGTCCAGCTCTGGCTCAATGGAACTCTGAGATTTAACAAGGAAAAAGACAAGGA
GCAGGATGCAGCTGAGTTCTCCTTTGCTGTCACAAACCTGGAGGACGCCGGGATATATCAGTGTCGGTACCAG
GTGTCAGAGCCACTGTGGACATCAAATCAGAGTGACCCCGTGGAG

>EM11T7-1
GCCTCAGCCGGCTGCCTGGGTCCGACTCTTTTGGTACCAAAATCCAACATACATCGAGCAGAAGGACAAAACG
CAGGAAATGGCAGAGTTCTCCTTCCGTAGCGCAAGTCATGATCAAGCAGGTACATATTTGTGCCAGTACCAGG
TGTCTGAGTCAGAGGACGTATCAGTGATGAGTGACCCTGTGGAG

>EM12T7-1
GCCCCGGCCGGCTGCCTGGGTCTGGCTGTATCTGGAAGGACGTTGGTCATACGCCAAGTACAGTGACAAGAA
GCAGGACACGACAGAGTTCTCCTTCCTTAGTGCAAGTCAGGATCAAGCAGGTACATATTTGTGCCAGTACCAG
GTGTCTGAGTCAGAGGACGTATCAGTGATGAGTGACCCCGTGGAG

>EM13T7-1
GCCCCAACTGGCTGCCTGGGTCTGGCTGTACCAGGAAGGAGGTAGGACACACAACAAGTACAAGGACAAGG
AGCAGGATGCAGTCGAGTTCTCTTTTGGTAACACAAGTTGGAAACATGCGGGGACGTATCGGTGCCATTACCA
TGTGTCTGAGCCACTGGGGACTTCAGAGAAGAGCGATCCTGTGGAG

>EM14T7-1
GCCCCGCGTGGATGCCTGGGTCCAGCTCTGGCACAATGGAACTCTGAGATTTAACAAGGAAAAAGACAAGGA
GCAGGATGCAGCTGAGTTCTCCTTTGCTGTCACAAACCTGGAGGACGCCGGGACGTATCAGTGTCGGTACCA
GGTGTCAAAGCCTCTGAGGACATCCAAAAAGAGTGACCCCGTGGAG

>EM15T7-1
GCCCCGCATGGCTGCCTGGGTCCAGCTCTGGCTCAATGGAACTCTGAGATTTGACAAGGAAAAAGACAAGGA
GCAGGATGCAGCTGAGTTCTCCTTTGCTGTCACAAACCTGGAGGACGCCGGGACATATCAGTGTCGGTACCA
GGTGTCAAAGCCTCTGAGGACATCCAAAAAGAGTGACCCCGTGGAG

>EM16T7-1
GCCCCGCATGGCTGCCTGGGTCCAGCTCTGGCACAATGGAACTCTGAGATTTAACAAGGAAAAAGACAAGGA
GCAGGATGCAGCTGAGTTCTCCTTTGCTGTCACAAACCTGGAGGACGCCGGGACATATCAGTGTCGGTACCA
GGTGTCAGAGCCACTGTGGACATCAAATCAGAGTGACCCCGTGGAG

>EM17T7-1
GCCCCAGTTGCACTTACAAGTTCAGCTCTGCCAGAACGGACATATGAGATCCAAGAAAGACGCGGAGATGCT
TCAGGACACAGTTGAGTTTTCCTTGGTTAGCGTAGAGAAGGAAGACGCAGAGAAGTATCGGTGCCAGTACCG
GGTTTTGGAGCCACCGGGGACATCAGGGAAGAGTGACCCTGTGGAG

>EM18T7-1
GCCCCAGCCAGCTGCCAGGGTCGAACTGTACCAGGAAGAAAAGTTGAGATCCAGCAAGGAAATGGACCAGG
AGCATGACGTGGCTGAGTTCTCCTTGGCTGGAATTAAGCAGGAAGATGCAGTGAGATATCAGTGCCAGTACC
AGGGGTTGGAGCCTGTAGGGACATCGGAGAAGAGTGACCCCGTGGAG

>EM19T7-1
GCCCCGCATGGCTGCCTGGGTCCAGCTCTGGCTCAATGGAACTCTGAGATTTAACGAGGAAAAAGACAAGGA
GCAGGATACAGCTGAGTTCTCCTTTGCTGTCACAAACCTGGAGGACGCCGGGACATATCAGTGTCGGTACCAG
GTGTCAGAGCCTCTGAGGACATCCAAAAAGAGTGACCCTGTGGAG

Fig. S1, continued page 2 of 4. Sequences of 39 clones of 190 base pairs from two independent amplifications of exon 2 from a line 6_1 chicken, plus the two FLRs

>EM20T7-1
GCCCCAGCCAGCTGCCTGGGTCTGGCTGTACCAGGAGGGAGGTTGGTCGTACAACAAGGGGAAGGAGAAGG
AGCAGGATGTGGCCGAGTTCTCCTTTGTTAGCACACTGCGGGAACACGCAGGTCGTTATCAGTGTCAGTACCG
GGTGTCATGGTCAGAAGAGGCATCAGAGAAGAGTGACCCCGTGGAG

>EM22T7-1
GCCCCGGCCAGCTACCAGGGTCGAGCTGTATCAGGATGGACAGTGGAGATCCAGAAAGGAAATGGACCAGG
AGCAGGACGTGGCTGAGTTCTCCTTGGCTGGAATAAAGAAGAAAGATTCGGGGACATATCAGTGCCAGTACC
AGGGGTTGGCACCTGCAGGGACATCAGAGAAGAGTGACCCCGTGGAG

>EM23T7-1
GCCCCAGCTGGCTGCCTGGGTCTGGCTGTACCAGGAAAGAGGTTGGTCATACAACAAGGGGAAGGAGAAGG
AGCAGGACGCGGCCGAGTTCTCCTTTGTTAGCACACAGCGGGAACACGCAGGTCGCTATCAGTGTCAGTACC
GGGAGTCTTGGTCAGTAGGGGCATCAGAGAAGAGTGACCCCGTGGAG

>EM24T7-1
GCCCCAGCCGGCTGCACAAGTTGAGCTCTACCAGAACGGACATATGAGATCCAAGAAAGACATGGACATGCT
ACAGAACACAGTTGAGTTTTCCTTGCTTAGTGTAGAGAAGGAAGATGCAGAGAAGTATCGGTGCCAGTACCG
GGTTTTGGAGCCACCAGGGATGTCAGGGAAGAGTGACCCCGTGGAG

>EM2T7-2
GCCCCGGTTAACCTTACAAGCTGAGCTCTGCCAGAACGGCCATTTGAGATCCAAGAAAGACATGGACAGACTT
CAGGACACGGTTGAGTTTTCCTTGGTTAGCGTAGAGAAGGAAGAGGCAGAGAAGTATCGGTGGCAGTACCG
GGTTTTGGAGCCACCGGGGACATCAGGGAAGAGTGACCCCGTGGAG

>EM4T7-2 curated
GCCCCGCATGGCTGCCTGGGTCCAGCTCTGGCACAATGGAACTCTGAGATTTGACAAGGAAAAAGACAAGGA
GCAGGATGCAGCTGAGTTCTCCTTTGCTGTCACAAACCTGGAGGACGCCGGGACATATCAGTGTCGGTACCA
GGTGTCAGAGCCACTGTGGACATCAAATCAGAGTGACCCCGTGGAG

>EM5T7-2
GCCCCGGCCAGCTACCAGGGTCGAGCTGTATCAGGATGGACAGTGGAGATCCAGAAAGGAAATGGACCAGG
AGCAGGACGTGGCTGAGTTCTCCTTGGCTGGAATAAAGAAGAAAGATTCGGGGACATATCAGTGCCAGTACC
AGGGGTTGGCACCTGCAGGGACATCAGAGAAGAGTGACCCCGTGGAG

>EM6T7-2 curated
GCCCCAGCTGGCTGCACAAGTTGAGCTCTACCAGAACGGACATATGAGATCCAAGAAAGACATGGACATGCT
ACAGGACACAGTTGAGTTTTCCTTGGTTAGTGTAGAGAAGGAAGATGCAGAGAAGTATCGGTGCCAGTACCG
GGTTTTAGAGCCACCAGGGATGCCAGGGAAGAGTGACCCCGTGGAG

>EM7T7-2
GCCCCAGCCAGCTGCCTGGGTCGAGCTGTACCAGGATGGACAGTTGAGATCCAGCAAGGAAATGGACCAGA
AGCAGGACGCGGTTGAGTTCTCCTTGGCTGGCATAAAGAAGGAAGATTCGGGGACATATCAGTGCCAGTACG
AGGGGTTGGAGCAACCAGGGACATCGGAGAAGAGTGACCCCGTGGAG

>EM9T7-2 curated
GCCCCACATGGCTGCCTGGGTCTGGTTGTACCACGAAGAAGGTCGGTCGTACAACAAAGGGAAGAAGAAGG
AGCAGGACGCGGCCGCGTTCTTTTTTGTTAGCACACTGCAGGAACACGCAGGTCGTTACTGGTGTCAGTACCG
GGTGTCTGAGTCAGCCGAGGTGTCAGTGAAGAGTGACCCTGTGGAG

Fig. S1, continued page 3 of 4. Sequences of 39 clones of 190 base pairs from two independent amplifications of exon 2 from a line 6_1 chicken, plus the two FLRs

>EM11T7-2
GCCCCAGTTGCACTTACAAGTTCAGCTCTGCCAGAACGGACATATGAGATCCAAGAAAGACGCGGAGATGCT
TCAGGACACAGTTGAGTTTTCCTTGGTTAGCGTAGAGAAGGAAGACGCAGAGAAGTATCGGTGCCAGTACCG
GGTTTTGGAGCCACCGGGGACATCAGGGAAGAGTGACCCCGTGGAG

>EM12T7-2
GCCCCGCATGGCTGCCTGGGTCTGGCTGTACCAGGAAAGAGGTTGGTCGTACAACAAGGGGAAGGAGAAGG
AGCAGAACGCGGCCGAGTTCTTCTTTGTTAGCACAAAGCGGGAACACGCAGGTCGTTATCGGTGTCAGTACC
GGGTGTCTTGGTCAGTAGGGGCATCAGAGAAGAGTGACCCCGTGGAG

>EM13T7-2 curated
GCCCCGGCCGGCTGCCTGGGTTGAGCTGTACTGGGAGGGCAAGTGGAGATTCAGAAAAGAAATGGACCAGG
AGCAGGACGTGGCTGAGTTCTCCTTGACTGGAATTAAGCAGGAAGATGCAGTGAGATATCAGTGCCAGTACC
AGGGGTTGGAGCCTGCAGGGACATCGCAGAAGAGTGACCCCGTGGAG

>EM14T7-2
GCCCCGGCCGGCTGCCTGGGTTGAGCTGTACAAGGAGGGAAAGTGGAGATCCAGAAAAGAAATGGACCAGG
AGCAGGACGTGGCTGAGTTCTCCTTGACTGGAATTAAGCAGGAAGATGCAGTGAGATATCAGTGCCAGTACG
AGGGGTTGGAGCAACCAGGGACATCGGAGAAGAGTGACCCCGTGGAG

>EM15T7-2
GCCCCACATGGCTGCCTGGGTCTGGTTGTACCACGAAGAAGGTCGGTCGTACAACAAAGGGAAGAAGAAGG
AGCAGGACGCGGCCGCGTTCTTTTTTGTTAGCACACTGCAGGAACACGCAGGTCGTTACTGGTGTCAGTACCG
GGTGTCTGAGTCAGCCGAGTTGTCAGTGGAGAGTGACCCCGTGGAG

>EM16T7-2 curated
GCCTCAGCCGGCTGCCTGGGTCCGACTCTTTTGGTACCAAAATCCAACATACATCGAGCAGAAGGACAAAACG
CAGGAAATGGCAGAGTTCTCCTTGACTGGAATTAAGCAGGTAGATGCAGTGAGATATCAGTGCCAGTACCAG
GGGTTGGAGCAACCAGGCACATCAAATCAGAGTAACCCCGTGGAG

>EM18T7-2 curated
GCCCTGGTTGCCCTTACAAGTTGAGCTCTGCCAGAACGGACATTTGAGATCCAAGAAAGACGTGGAGATGCTT
CAGGACACGGTTGAGTTTTCCTTGGTTAGCCTAGAGAAGGAAGACGCAGAGAAGTATCGGTGCCAGTACCGG
GTTTTGGAGCCACCAGGGACATCAGGGAAGAGTGACCCCGTGGAG

>EM20T7-2 curated
GCCCCAGCCGGCTGCACAAGTTGAGCTCTACCAGAACGGACATATGAGATCCAAGAAAGACATGGACATGCT
ACAGAACACAGTTGAGTTTTCCTTGCTTAGTGTAGAGAAGGAAGATGCAGAGAAGTATCGGTGCCTGTACCG
GGTTTTGGAGCCACCAGGGATGTCAGGGAAGAGTGACCCCGTGGAG

>EM21T7-2 curated
GCCCCGGCCAGCTACCAGGGTCGAGCTGTATCAGGATGGACAGTGGAGATCCAGAAAGGAAATGGACCAGG
AGCAGGACGTGGCTGAGTTCTCCTTGGCTGGAATAAAGAAGAAAGATTCGGGGACATATCAGTGCCAGTACC
AGGGGTTGGCACCTGCAGGGACATCAGAGAAGAGTGACCCCGTGGAG

Fig. S1, continued page 4 of 4. Sequences of 39 clones of 190 base pairs from two independent amplifications of exon 2 from a line 6_1 chicken, plus the two FLRs

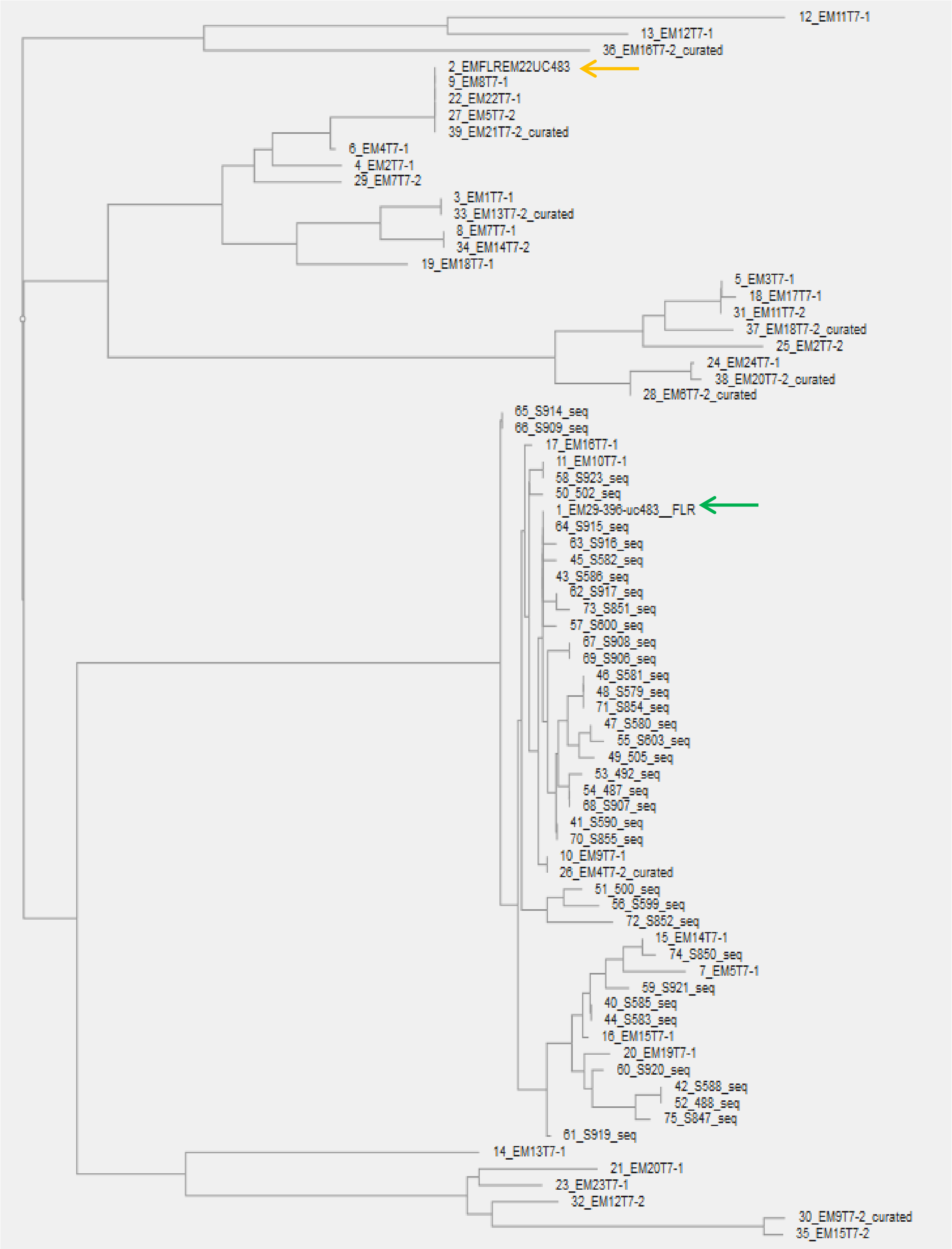

Fig. S2. Neighbour joining tree of 36 cloned sequences from Munich lines M11 and R11 (all indicated by names starting with S), and 39 sequences from a line 6_1 chicken (all indicated by names starting with EM), plus the two FLRs (indicated by arrows, orange for FLR22 and green for FLR29). The tree was constructed using MAFFT (https://mafft.cbrc.jp/alignment/server/) displayed using Archaeopteryx.js available on the same website.

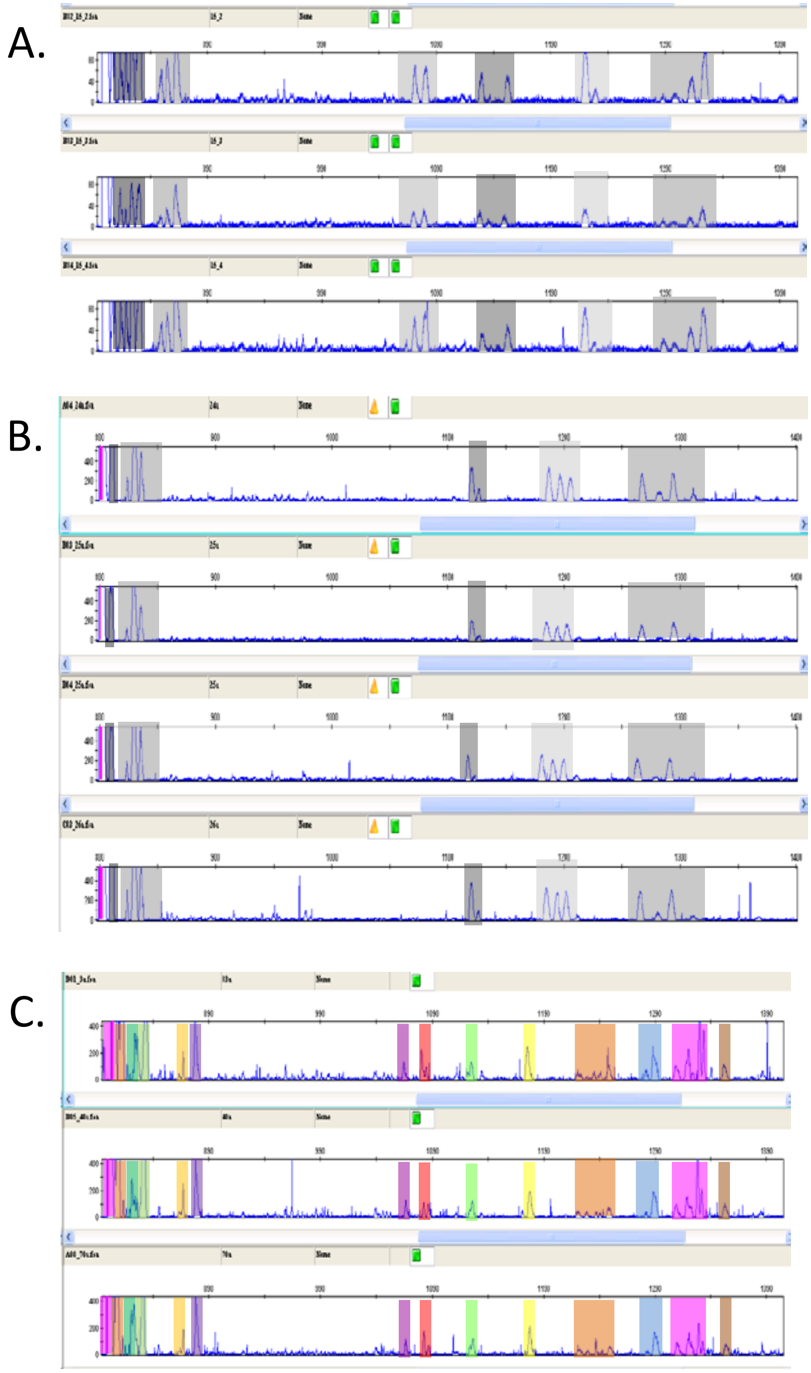

Fig. S3. RSCA of ChIR-AB1 amplicons using FLR29 show that patterns can be very reproducible between different individuals within a class of patterns on the same run.

A. three individuals from line 15I (pattern 2),

B. B. four individuals from line C-B4 (pattern 4c),

C. C. three individuals from the first commercial broiler line

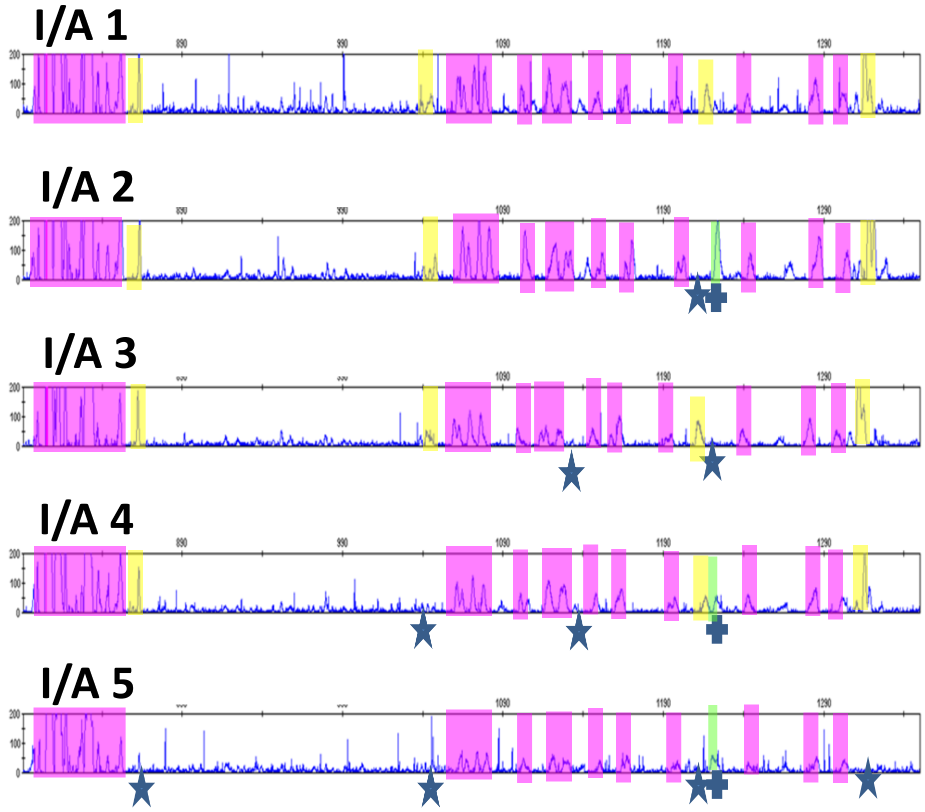

Fig. S4. RSCA of ChIR-AB1 amplicons from one group of commercial broiler chickens using FLR29 shows peaks that appear or disappear. Purple highlight indicates peaks common to all patterns shown, yellow highlight and star indicate peaks shared with pattern I/A-1, green highlight and plus sign indicate peaks not shared with pattern I/A-1.

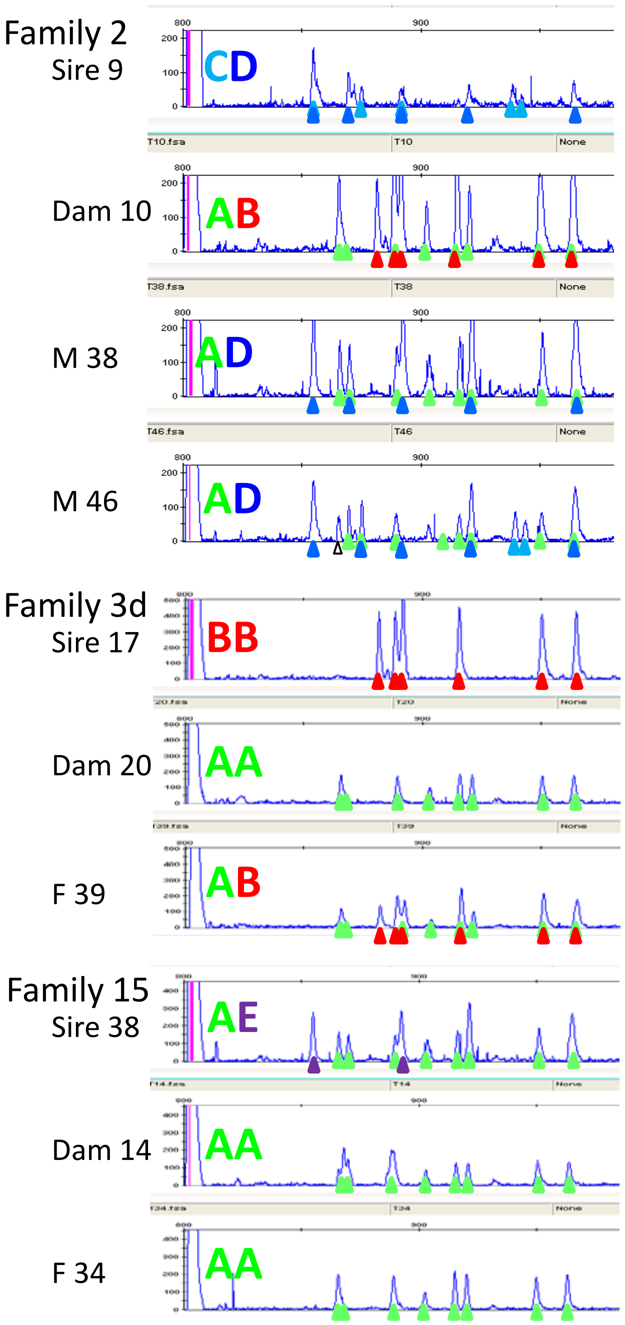

Fig. S5. Families (trios) in a line of commercial birds allow haplotypes to be discerned by analysis of RSCA patterns using FLR22. Letters indicate haplotypes with colours of triangles indicating peaks assigned to that haplotype. Page 1 of 2.

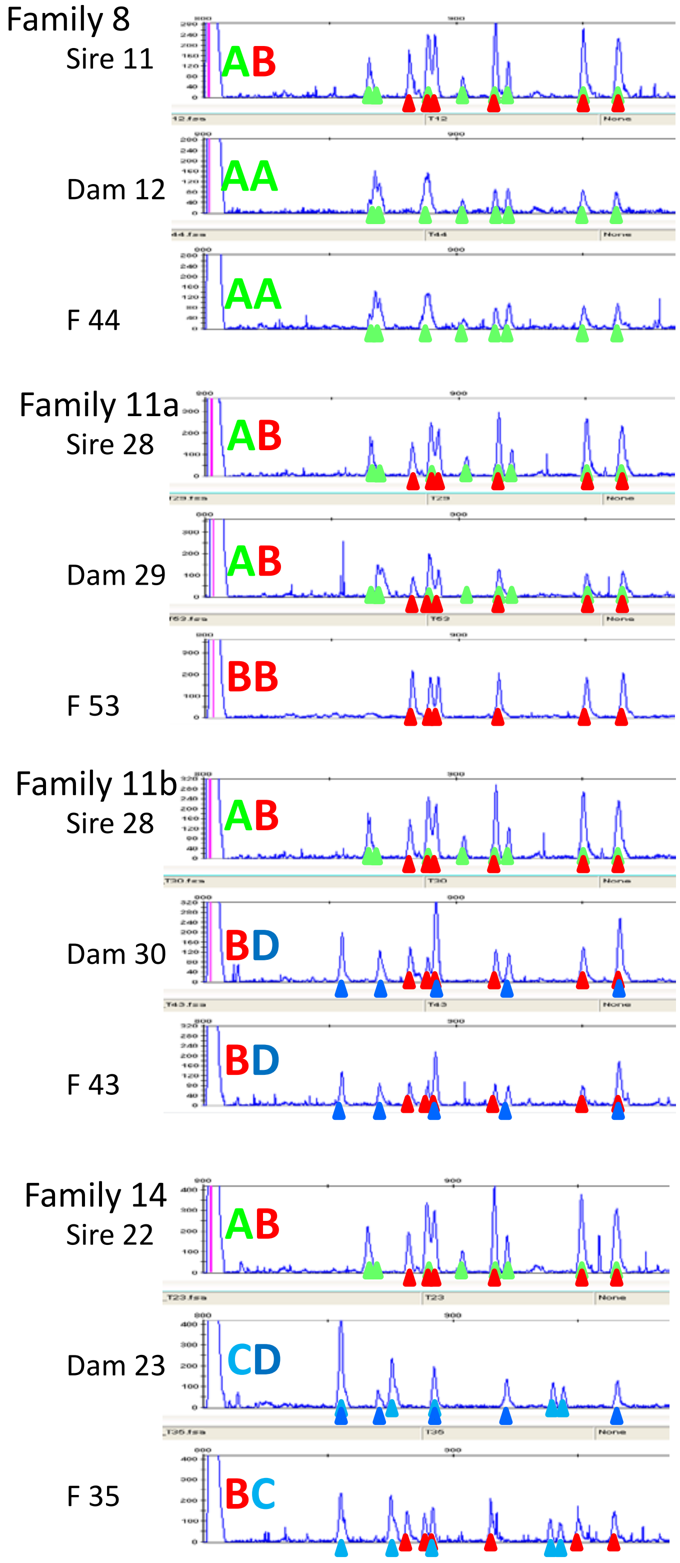

Fig. S5, continued. Families (trios) in a line of commercial birds allow haplotypes to be discerned by analysis of RSCA patterns using FLR22. Letters indicate haplotypes with colours of triangles indicating peaks assigned to that haplotype. Page 2 of 2.

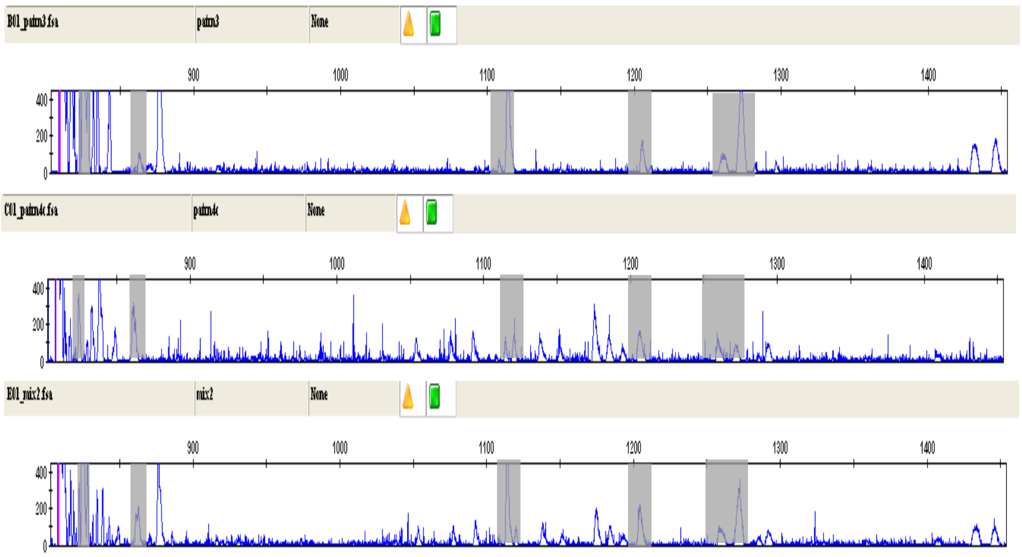

Fig. S6. RSCA with mixed samples identifies peaks with the same mobility, confirming identical peaks. Two samples with different peak patterns (3 and 4c) were analysed either separately (top two panels) or mixed together (half and half to give the same loading volume, bottom panel). The shaded peaks that appear to have the same mobility when analysed separately are confirmed to collapse to a single peak, while other peaks remain unchanged.
